# Unexpected rapid increase in bone mineral density by bisphosphonate therapy after multiple spinal fractures: a case report

**DOI:** 10.1186/s13256-019-2219-0

**Published:** 2019-09-13

**Authors:** Hidefumi Koiwai, Mikio Kamimura, Jun Takahashi, Yukio Nakamura, Hiroyuki Kato

**Affiliations:** 1 Koiwai Orthopedic Clinic, Mikageshinden 1585-4, Komoro, 384-0091 Japan; 2 Center of Osteoporosis and Spinal Disorders, Kamimura Orthopedic Clinic, Kotobuki 595-17, Matsumoto, 399-0021 Japan; 3 Department of Orthopaedic Surgery, Shinshu University School of Medicine, Asahi 3-1-1, Matsumoto, 390-8621 Japan; 4 Department of Orthopedic Surgery, Showa-Inan General Hospital, Akaho 3230, Komagane, 399-4117 Japan

**Keywords:** Bone mineral density, Bisphosphonate, Osteoporosis, Spinal fracture

## Abstract

**Background:**

Osteoporosis is a serious disease that causes bone fragility fractures and increases mortality. Bisphosphonates are the first-line drugs for osteoporosis. However, the gains in bone mineral density by use of bisphosphonates alone are limited.

**Case presentation:**

We describe the clinical outcome of a Japanese woman with osteoporosis treated with bisphosphonates after multiple spinal fractures. After 3 years of treatment with the bisphosphonate alendronate, her lumbar bone mineral density and bilateral hip bone mineral density markedly increased by 61.9% and 32.5%, respectively.

**Conclusion:**

We considered that our patient’s multiple fractures had caused a decrease in bone mineral density, which naturally improved with fracture healing to enhance the increase in bone mineral density with bisphosphonate treatment.

## Introduction

Osteoporosis (OP) is a serious and widespread disease that predisposes patients to bone fragility fractures and increases mortality. Bisphosphonates (BPs) are the first-line drugs for OP treatment to increase bone mineral density (BMD) and prevent fractures [1, 2]. However, the improvements in BMD by BPs alone are limited and gradually diminish after the first few years [3, 4]. Bone *et al.* observed that alendronate (ALN) provided increases of 13.8% in lumbar spine BMD (L-BMD) and 7.8% in total hip BMD (H-BMD) during 10 years [4]. Compared with BPs, teriparatide (TPTD) (parathyroid hormone 1–34) has stronger effects on L-BMD increase and inhibition of spinal fractures [5]. Therefore, TPTD may be preferential for patients with OP who have greatly diminished BMD and/or multiple bone fractures. We recently encountered a rare case of a patient with OP in whom BMD increased remarkably with BP treatment only.

## Case presentation

Our patient was a 59-year-old Japanese woman 49 kg in weight and 153.2 cm in height. Her chief complaint upon presentation was severe back pain. She reported having a total of five episodes of strong acute back pain in the previous year.

At the first visit, her L-BMD was 0.572 g/cm^2^ (− 4.5 standard deviation [SD]), and her H-BMD was 0.671 g/cm^2^ (− 2.2 SD). Spinal radiographs showed five vertebral compression fractures. Bone turnover markers (BTMs) were highly increased (Table 1). Bone scintigraphy revealed high accumulation at one lumbar vertebra and one thoracic vertebra, with diffuse mild uptake throughout the entire spine.

**Table 1:** Changes in laboratory values during bisphosphonate therapy

	Pretreatment	2 Months	4 Months	24 Months
ALP (U/L)	827	753	367	197
NTX (nmol BCE/mmol Cr)	313.5	9.8	31.3	13.9
BAP (μg/L)	90.5	94.7	32.3	12.7

*ALP* Alkaline phosphatase, *BAP* Bone alkaline phosphatase, *BCE* Bone collagen equivalents, *Cr* Creatinine, *NTX* Urinary N-terminal telopeptide of type I collagen

**Fig. 1:**
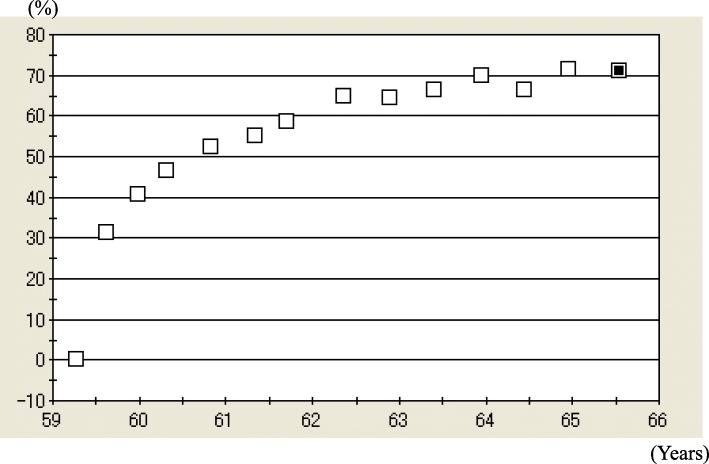
Percentage changes in lumbar 1–4 bone mineral density (L-BMD) from 59 to 66 years of age. L-BMD was 0.574 g/cm^2^ (− 4.5 standard deviation [SD]) prior to treatment, 0.824 g/cm^2^ (− 2.4 SD; 44.7% increase) at 1 year, 0.877 g/cm^2^ (− 1.9 SD; 53.3% increase) at 2 years, 0.926 g/cm^2^ (− 1.5 SD; 61.9% increase) at 3 years, and 0.957 g/cm^2^ (− 1.3 SD; 66.3% increase) at 6.5 years

**Fig. 2:**
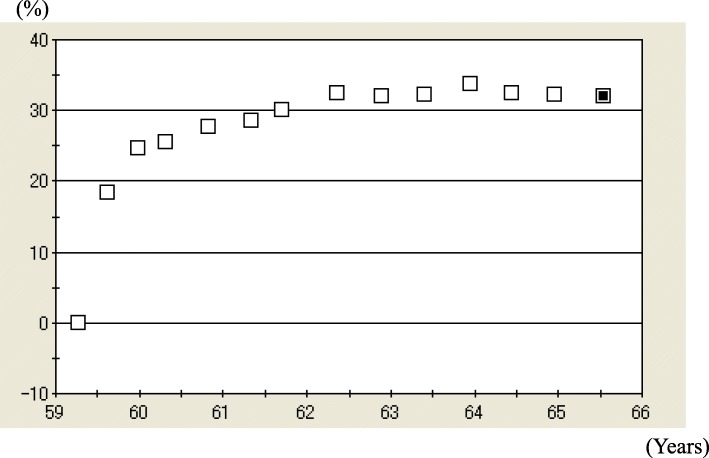
Percentage changes in bilateral total hip bone mineral density (H-BMD) from 59 to 66 years of age. H-BMD was 0.671 g/cm^2^ (− 2.2 standard deviation [SD]) prior to treatment, 0.842 g/cm^2^ (− 0.8 SD; 25.5% increase) at 1 year, 0.862 g/cm^2^ (− 0.6 SD; 28.5% increase) at 2 years, 0.889 g/cm^2^ (− 0.4 SD; 32.5% increase) at 3 years, and 0.885 g/cm^2^ (− 0.4 SD; 31.9% increase) at 6.5 years

She was diagnosed with OP in accordance with the revised criteria established by the Japanese Society for Bone and Mineral Research [6]. Because daily TPTD was not possible, owing to her living abroad, ALN 35 mg/week was prescribed until her planned return. One month later, her back pain was relieved, and she returned home. At 4 months of ALN, L-BMD and H-BMD were greatly increased by 29.5% and 18.3%, respectively (Figs. 1, 2). Hence, ALN treatment was continued.

The patient’s BTMs began to decrease soon after therapy commencement (Table 1). During 2 years of treatment, the patient’s urinary N-terminal telopeptide of type I collagen (NTX) decreased by 95.6% and serum bone alkaline phosphatase (BAP) fell by 85.6%. At 3 years of ALN monotherapy, L-BMD and H-BMD were markedly increased by 61.9% and 32.5%, respectively (Figs. 1, 2), which remained high over a treatment period of 6.5 years.

The present report was approved by the Institutional Ethics Committee at Shinshu University School of Medicine. The patient gave written informed consent for publication of her personal medical information prior to the start of treatment.

## Discussion

After the first vertebral fracture, the risk of subsequent fractures increases greatly and may occur consecutively in a so-called vertebral fracture cascade [7]. The patient had experienced severe acute back pain five times throughout the year preceding treatment. Spinal radiographs confirmed five vertebral compression fractures, indicating the occurrence of a fracture cascade.

BTMs are normally increased during the fracture-healing process [8, 9]. At the first visit, our patient’s BTMs were extremely high (BAP, 90.5 μg/L; NTX, 313.5 nmol bone collagen equivalents/mmol Cr). However, they soon decreased after treatment commencement and were within reference values 2 years later. Thus, the BTMs might have been greatly increased by the vertebral fractures and then improved both from fracture healing and from ALN.

The effects of BPs on BMD are potent but limited. In a phase III study in Japan, the increases of L-BMD were 6.2% for ALN 5 mg/day [10] and 4.9% for risedronate 2.5 mg/day [11] at 48 weeks. L-BMD was also increased by 9.2% at 3 years of treatment with ALN 5 mg/day [12]. In our patient’s case, BMD increased remarkably with ALN alone during 3 years (L-BMD, 61.9%; H-BMD, 32.5%).This rate of increase is unexpected and supports an additional mechanism of BMD improvement, such as the natural healing of the fracture cascade.

We previously reported two cases of pregnancy- and lactation-associated OP with multiple vertebral fractures [13]. In both patients, BMD gains with vitamins D and K treatment were impressive at 36.1% at 4 years in the first patient and 26.3% at 3 years in the second. In a recently encountered patient with anorexia nervosa, BTMs were highly increased by multiple vertebral fractures [14]. Treatment using a vitamin D analogue alone achieved remarkable L-BMD and H-BMD gains of 70.7% and 41.4%, respectively, at 8 months, although BMD improvement by vitamin D and/or K is usually very small. Similarly, in the present case, BMD increased markedly with BP monotherapy, with L-BMD gains of 29.5% at 4 months and 48.9% at 1 year. Results such as these seem possible only with simultaneous healing of fractures.

The common point in the above reports is the benefit of OP treatment after multiple fractures. On the basis of our findings, it was considered that multiple fractures might have caused decreases in BMD, which might be naturally improved by fracture healing to enhance the effects of OP treatment.

### Limitations

The lack of serum 25(OH)D_3_ data is a major limitation of this study. We have previously reported that 4 months of BP therapy significantly decreased 25(OH)D_3_ and that 3-year BP therapy without vitamin D supplementation significantly increased 25(OH)D_3_ [15, 16]. However, in our patient’s case, BMD rapidly increased; thus, we speculated that 25(OH)D_3_ levels might not have had an obvious influence on the results in this study. Another limitation of the present study includes its retrospective design. Nonetheless, our case report provides evidence of a good response in BMD after BP treatment in a patient with multiple fractures.

## Conclusions

We report a rare case, that of a 59-year-old woman with OP in whom BMD increased remarkably with BP treatment only. This case report reveals that multiple fractures might have caused decreases in BMD, which were naturally improved by fracture healing to enhance the effects of OP treatment.
